# Petunia Performance Under Application of Animal-Based Protein Hydrolysates: Effects on Visual Quality, Biomass, Nutrient Content, Root Morphology, and Gas Exchange

**DOI:** 10.3389/fpls.2021.640608

**Published:** 2021-06-14

**Authors:** Giuseppe Cristiano, Barbara De Lucia

**Affiliations:** Department of Agricultural and Environmental Sciences, University of Bari Aldo Moro, Bari, Italy

**Keywords:** biostimulant, environmentally friendly ornamentals, foliar spray, marketable extra grade, pot plant

## Abstract

Sustainable plant production practices have been implemented to reduce the use of synthetic fertilizers and other agrochemicals. One way to reduce fertilizer use without negatively impacting plant nutrition is to enhance crop uptake of nutrients with biostimulants. As the effectiveness of a biostimulant can depend on the origin, species, dose, and application method, the aim of this research was to evaluate the effect of a commercial animal-based protein hydrolysate (PH) biostimulant on the visual quality, biomass, macronutrient content, root morphology, and leaf gas exchange of a petunia (*Petunia* × *hybrida* Hort. “red”) under preharvest conditions. Two treatments were compared: (a) three doses of an animal-based PH biostimulant: 0 (D0 = control), 0.1 (D0.1 = normal), and 0.2 g L^–1^ (D0.2 = high); (b) two biostimulant application methods: foliar spray and root drenching. The dose × method interaction effect of PH biostimulant on the plants was significant in terms of quality grade and fresh and dry biomass. The high dose applied as foliar spray produced petunias with extra-grade visual quality (number of flowers per plant 161, number of leaves per plant 450, and leaf area per plant 1,487 cm^2^) and a total aboveground dry weight of 35 g, shoots (+91%), flowers (+230%), and leaf fresh weight (+71%). P and K contents were higher than in untreated petunias, when plants were grown with D0.2 and foliar spray. With foliar spray at the two doses, SPAD showed a linear increase (+21.6 and +41.0%) with respect to untreated plants. The dose × method interaction effect of biostimulant application was significant for root length, projected and total root surface area, and number of root tips, forks, and crossings. Concerning leaf gas exchange parameters, applying the biostimulant at both doses as foliar spray resulted in a significant improvement in net photosynthesis (D0.1: 22.9 μmol CO_2_ m^–2^ s^–1^ and D0.2: 22.4 μmol CO_2_ m^–2^ s^–1^) and stomatal conductance (D0.1: 0.42 mmol H_2_O m^–2^ s^–1^ and D0.2: 0.39 mmol H_2_O m^–2^ s^–1^) compared to control. These results indicate that application of PH biostimulant at 0.2 g L^–1^ as foliar spray helped to achieve extra-grade plants and that this practice can be exploited in sustainable greenhouse conditions for commercial production of petunia.

## Introduction

The use of chemicals, water, energy, and plastic has exposed greenhouse horticulture to criticism for its environmental impact (Wandl and Haberl, 2017; Gruda et al., 2019). The marketability of greenhouse bedding plants is greatly influenced by the intensive conditions of their production, aimed at avoiding aesthetic defects due to nutritional imbalances and biotic and abiotic stresses.

Petunia (*Petunia* × *hybrida* Hort.) is a leading cultivated bedding plant used in private and public parks and gardens (Arancon et al., 2008); vegetatively vigorous petunias, such as Potunia^®^, with round habit and large flowers have revolutionized the genus. Nurserymen grow petunias in limited pot volumes that require frequent irrigation and high fertilization rates (James and van Lersel, 2001), ranging from 200 to 500 mg L^–1^ N (Chavez et al., 2008; Fain et al., 2008), which can cause contamination of ground and surface water (Lang and Pannuk, 1998; Hansen et al., 2017; Shu et al., 2019) and climate change (Bouwman et al., 2013; Zhang et al., 2015). Today, it is necessary to consider the sustainability of nursery production (Isaak and Lentz, 2020). Sustainable plant production practices have been studied to reduce the use of synthetic fertilizers and other agrochemicals. One way in which fertilizer use can be reduced without negatively impacting plant nutrition is to enhance crop uptake of nutrients with biostimulants (Kunicki et al., 2010; Baglieri et al., 2014; Halpern et al., 2015; De Pascale et al., 2017; Toscano et al., 2018; Parađiković et al., 2019; De Pascale et al., 2020). “A plant biostimulant shall be an EU fertilizing product, the function of which is to stimulate plant nutrition processes independently of the product’s nutrient content with the sole aim of improving one or more of the following characteristics of the plant or the plant rhizosphere: (i) nutrient use efficiency, (ii) tolerance to abiotic stress, (iii) quality traits, or (iv) availability of confined nutrients in the soil or rhizosphere” (The European Parliament and the Council of the European Union, 2019).

Protein hydrolysates (PHs) consisting mainly of signaling peptides and free amino acids, “manufactured from protein sources by partial hydrolysis” (Schaafsma, 2009), have gained prominence as non-microbial biostimulants because of their potential to enhance plant growth, yield, and quality (Ertani et al., 2009; Calvo et al., 2014; Colla et al., 2014, 2017a,b; Nardi et al., 2016; Carillo et al., 2019; Rouphael and Colla, 2020). Venugopal (2016) found that enzymatic hydrolysis of plant or animal sources ensures biostimulant products of higher quality than does chemical hydrolysis. Animal-based PH biostimulants have a higher N content (9–16% d.m.) than plant-based biostimulants (Colla et al., 2015).

Vegetables, such as tomato (Polo and Mata, 2018; Sestili et al., 2018; Casadesús et al., 2019), rocket (Caruso et al., 2019), celery (Consentino et al., 2020), lettuce (Polo et al., 2006; Xu and Mou, 2017), basil (Rouphael et al., 2021), and spinach (Kunicki et al., 2010), and tree crops, such as kiwifruit (Quartieri et al., 2002), papaya (Morales-Pajan and Stall, 2003), and passion fruit (Morales-Pajan and Stall, 2004), have been tested with animal-based PHs, with the aim of improving plant performance and abiotic stress resistance. Less attention to the use of animal-based PH biostimulants has been paid for ornamental species, especially bedding plants. In a globalized world, consumer demand for quality and novelty guides the global market of ornamental bedding plants (Lütken et al., 2010). Regarding consumers, a study carried out by Sánchez-Bravo et al. (2021) showed that a sustainable product has better quality. Sustainability is achieved via critical adjustments on cultivation by minimizing fuel and electricity use, adopting integrated nutrient management and integrated pest and disease management, and using recyclable materials and peat-alternative growing compounds (European Biostimulants Industry Council (EBIC), 2013; Darras, 2020). Sustainability assessment of potted plant focused mainly on environmental aspects such as carbon footprint (Soode et al., 2013; Ingram et al., 2019; Havardi-Burger et al., 2020).

If we apply the concept of quality of vegetable seedlings (Gruda, 2005) to ornamental plants, we can say that quality is not fixed but is a complex prerequisite. Quality has various extrinsic, or visual, and intrinsic, such as environmental and social, components. Consumers choose flowering plants with high aesthetic quality: compact, branched, with many flowers and leaves, a good balance between plant and pot size, and dark green leaves without blemishes or signs of stress (Kader, 2000; Ferrante et al., 2015; Bergstrand, 2017). Flower grading means dividing flowers into several grades according to the quality based on the appearance (Sun et al., 2017). For marketing purposes, ornamental potted plant quality is classified into four grades according to appearance: extra (extra-large) > 1st (large) > 2nd (medium) > 3rd (small)^1^.

Unfortunately, for bedding plants, few growers and traders pay attention to quality grading.

The visual quality of ornamental plants is necessarily linked to an adequate content of nutrients, in order to achieve the standards of commercialization and consumption (Marschner, 2011). Nitrogen (N) is the chief among minerals in plant nutrition, and its deficiency is considered as one of the limiting factors for quality: Nordstedt et al. (2020) showed that the visual symptoms of leaf yellowing in control plants compared to biostimulant-treated plants (*Pseudomonas* strains) were less severe in *P.* × *hybrida*, increasing the quality of ornamental potted plants grown under low-nutrient regimens.

Leaf gas exchanges can be used for evaluating the efficacy of biostimulant treatments: Bulgari et al. (2019) on lettuce reported that the biostimulant Retrosal^®^ could stimulate crop performance and quality by keeping open stomata, maintaining photosynthesis, source-sink relations (growth), and thus protecting from possible photoinhibition/photo-oxidation effects.

Therefore, a comprehensive study is needed to improving quality and sustainability in potted petunia cultivation.

As the effectiveness of a biostimulant can depend on origin, species, dose, and application method, the aim of this research was to evaluate the effect of a commercial animal-based PH biostimulant on the quality, biomass, macronutrient content, root morphology, and leaf gas exchange of petunia under preharvest conditions.

## Materials and Methods

### Treatments and Experimental Design

Two treatments were compared: (a) three doses of an animal-based PH biostimulant (D): 0 (D0 = control), 0.1 (D0.1 = normal), and 0.2 g L^–1^ (D0.2 = high); and (b) two biostimulant application methods (M): foliar spray (Fo) and root drenching (Dr).

The biostimulant was applied to the leaves of petunias, using a manual sprayer at a volume of 150 mL plant^–1^. Root drenching was performed applying the same volume directly to the growing medium. The same volume of distilled water was applied to the control as foliar spray or root drenching.

The treatments were performed in randomized complete block design and 18 experimental units (three doses × two biostimulant application methods × three replicates). Each experimental unit consisted of 10 plants (*n* = 180 plants in total).

The experiment was conducted from November 2016 to March 2017 in the heated greenhouse at the University Campus in Bari (Italy) (41 07′33.79″ N; 16°52′09.44″ E; altitude 3.35 m); average air temperature was 20°C/13°C day/night, and relative humidity range was 40–65%. Rooted plants of *Petunia* × *hybrida* Hort., Potunia^®^ series, and red (Dunnen^®^) cultivar were used for the study. The Potunia^®^ series of petunias features vigorous, rounded, compact, well-branched plants with an abundance of flowers.

On November 10, 2016, single petunia plants were transplanted into 1.2-L pots that were arranged at a density of 15 plants m^–2^. The substrate was a mixture of peat (Plantaflor^®^, Germany) and perlite (Perlitech, Italy) 80:20 (vol/vol).

Hydrostim^®^ (Hydrofert, Italy), a completely soluble commercial animal-derived powdered PH product, authorized in organic farming, was used to treat the plants. It is obtained by enzymatic hydrolysis of proteins from erythrocytes (red blood cells) and contains 38% organic matter, 10.2% total N, and 52% amino acids (Table 1). The recommended dose is 10–12 g 100 L^–1^ for vegetables and trees and 15 g 100 L^–1^ for citrus trees.

**TABLE 1:** Amino acid content of the commercial animal-based PH biostimulant (Hydrostim) used on petunia plants.

Amino acid	Content (mg L^–^^1^)	Amino acid	Content (mg L^–^^1^)
Valine	0.15	Betaine	2.02
Threonine	0.34	Leucine	2.21
Tyrosine	0.34	Arginine	2.98
Methionine	0.38	Aspartic acid	3.45
Cysteine + cystine	0.46	Alanine	4.94
Isoleucine	0.86	Hydroxyproline	5.28
Phenylalanine	1.24	Proline	6.50
Lysine	1.85	Glutamic acid	6.52
Serine	1.62	Glycine	10.9
Histidine	<LQ	Tryptophan	<DL

*DL, detection limit.*

Biostimulant treatments began 4 weeks after transplant and were applied weekly eight times, until flower bud differentiation. The plants were fertigated with a nutrient solution used in the standard cultivation technique, containing (in mg L^–1^) 40 N, 8 phosphorus (P), 60 potassium (K), 44 calcium, and 8 magnesium, plus microelements (3 iron, 2 manganese, 0.1 copper, and 0.5 boron), E.C. 1.2 dS m^–1^, pH 5.8.

### Growth Measurements: Ornamental Visual Characteristics and Biomass

At harvest, 150 days after transplant, the plants were graded for visual quality and plant biomass. Leaf macronutrient content, root morphology, and gas exchange were also evaluated.

To determine visual quality and fresh and dry biomass, the plants were divided into four grades according to UE market rules: extra (extra-large) > 1st (large) > 2nd (medium) > 3rd (small) as reported in Table 2. All grades of Petunia plants are required to have the following characteristics, under penalty of rejection: symmetrical shape, optimum floral display, uniformly distributed flower buds, strong stems, verdant foliage, no evidence of nutritional deficiency, disease, insect damage or mechanical injury, and well-developed root system.

**TABLE 2:** Parameters and ranges of the four quality visual grades for petunia plants according to https://www.flowerscanadagrowers.com/uploads/2016/11/grades %20&%20standards%20for%20foliage%20plants1.pdf.

Quality visual grades	Flower/plant (no.)	Leaves/plant (no.)	Leaf area/plant (cm^2^)	Shoots fresh weight/plant (g)	Flowers fresh weight/plant (g)	Leaves fresh weight/plant (g)	Aboveground dry weight/plant (g)
Extra	143–165	417–451	1,345–1,489	167–193	37–44	75–84	32–37
First	120–142	382–416	1,199–1,344	140–166	29–36	66–75	27–31
Second	97–141	347–381	1,053–1,198	113–139	21–28	56–65	21–26
Third	74–96	312–346	907–1,052	86–112	13–20	46–55	15–20

To determine agronomic characteristics, the plants were separated from the growing medium and divided into shoots, leaves, and flowers. These were oven-dried to constant weight at 70°C. For each treatment, six plants were used to determine the number of shoots, leaves, and flowers per plant. Total leaf area per plant was measured with a leaf area meter (Delta-T; Decagon Devices, Pullman, WA, United States). Chlorophyll SPAD index (Minolta Chlorophyll Meter SPAD-502) and total aboveground (shoot + leaves + flowers) fresh and dry weight were also measured.

### Root Morphology

Root morphology was assessed on the basis of root length, projected and total surface area, and number of tips, forks, and crossings on six plants for each treatment. The root system was separated from the aerial part and substrate. It was washed and scanned at 400 dpi (Epson Expression© 10000 XL scanner; Japan). The images were then processed using image analysis software (WinRHIZO v. 2005b©; Regent Instruments Inc., QC, Canada).

### Leaf Nutritional Status

Nutrient concentrations were determined in leaf samples. N was analyzed by the Kjeldahl method; total P and K contents were quantified according to EN 13650 (2001) by ICP-OES (inductively coupled plasma–optical emission spectrometry). The results are expressed as percentage of macronutrients. Six plants were used for each treatment.

### Gas Exchange and Chlorophyll Fluorescence Measurements

At the phenological stage of full bloom, leaf gas exchange was measured using an IRGA LI-6400XT portable gas exchange system (Li-COR, Lincoln, NE, United States), equipped with a 2 cm^2^ leaf chamber with a built-in fluorescence system (LI-6400-40; Li-COR).

Input airflow and CO_2_ concentration were set at 300 μmol s^–1^, and CO_2_ concentration was fixed at 400 ppm, respectively. Measurements were performed at the same time of the day (9–11 am and 1–3 pm CET) to minimize physiological changes due to environmental effects on fully expanded mature leaves of the same age. The fluorescence measurements were performed on the plants using a different order each day. No shift in parameters was noted during the day as we avoided the early and late hours. The plants were never under water stress.

Leaves were exposed to a saturating photosynthetic photon flux density of 1,000 μmol m^–2^ s^–1^ at a temperature of 25°C and with relative humidity in the leaf cuvette in the range of 40–60%. The parameters were recorded when the leaves inside the chamber reached steady state. The instrument provides a continuous display of gas exchange parameters. Steady state was reached when the first decimal of photosynthesis was stable (and therefore the other parameters). This usually happened after 2–3 min, as the air flow of 0.44 L min^–1^ was sufficient to produce fast air turnover inside the small fluorescence chamber.

Photosynthesis (A) and stomatal conductance (gs) was calculated by Li-COR software. The maximum quantum efficiency of PSII (Fv/Fm) and the actual quantum yield of PSII in illuminated leaves (F′v/F′m) were measured following a saturating pulse of light (10,000 μmol m^–2^ s^–1^). The gas exchange and fluorescence data are means of at least eight leaves per replication. Fv/Fm determinations were performed after adapting the leaves to the dark for 30 min. Shading clips were used on measured leaves, and the plant to be measured was also placed in a dark room.

### Statistical Analysis

The data were analyzed by two-way analysis of variance using Co-Stat statistics software. Treatment means were separated by Duncan multiple-range test (*P* ≤ 0.05).

## Results

### Visual Quality Characteristics and Plant Biomass

**FIGURE 1:**
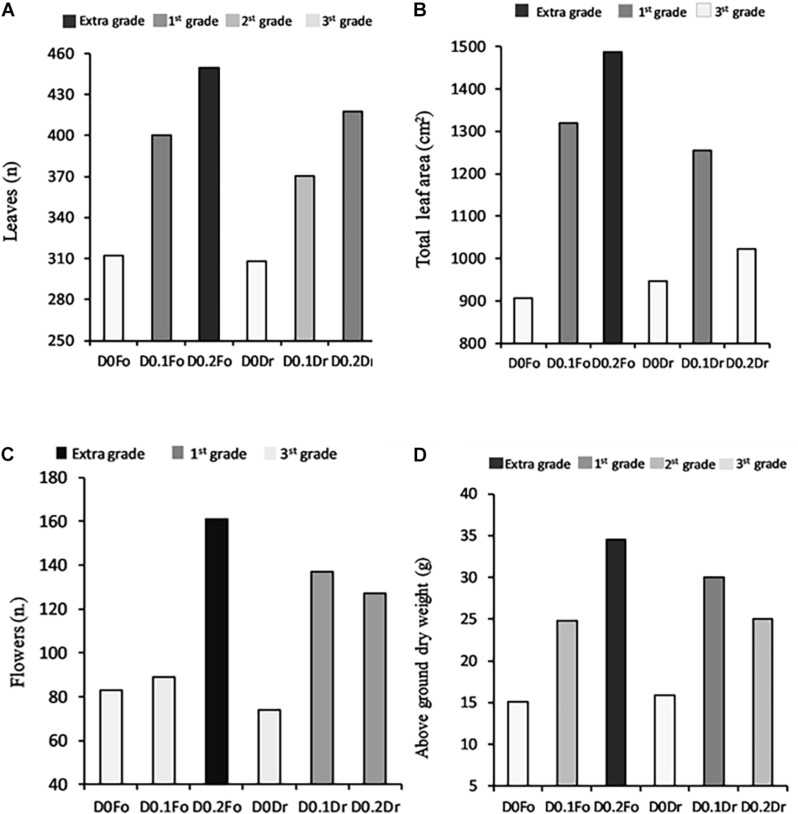
Interaction effects of biostimulant dose (D0, D0.1, and D0.2) × application method (Fo = foliar spray and Rd = root drenching) on petunia plant grade [leaf number **(A)**, total leaf area **(B)**, flower number **(C)**, and dry weight of aboveground parts **(D)**].

The dose × method (D × M) interaction effect of PH biostimulant and the plants was significant in terms of quality grade and dry biomass (Figure 1). Application of PHs to the plants at both doses (D0.1 and D0.2) increased quality grade with respect to untreated plants.

The high dose (D0.2) applied as foliar spray produced petunias with extra-grade visual quality (number of flowers per plant 161; number of leaves per plant 450; and leaf area per plant 1,487 cm^2^) and a total aboveground dry weight of 35 g.

Regarding the D × M interaction of biostimulant application, increasing PH concentration of foliar spray from D0.1 to D0.2 resulted in the best quality grade: 3rd grade (control) < 1st grade (D0.1) < extra grade (D0.2) for leaf area and aboveground dry biomass (Figure 1). Plants treated by root drenching at D0.1 achieved 1st grade, whereas controls achieved 3rd grade; application of biostimulant at D0.2 did not result in any significant improvement in quality grade.

**FIGURE 2:**
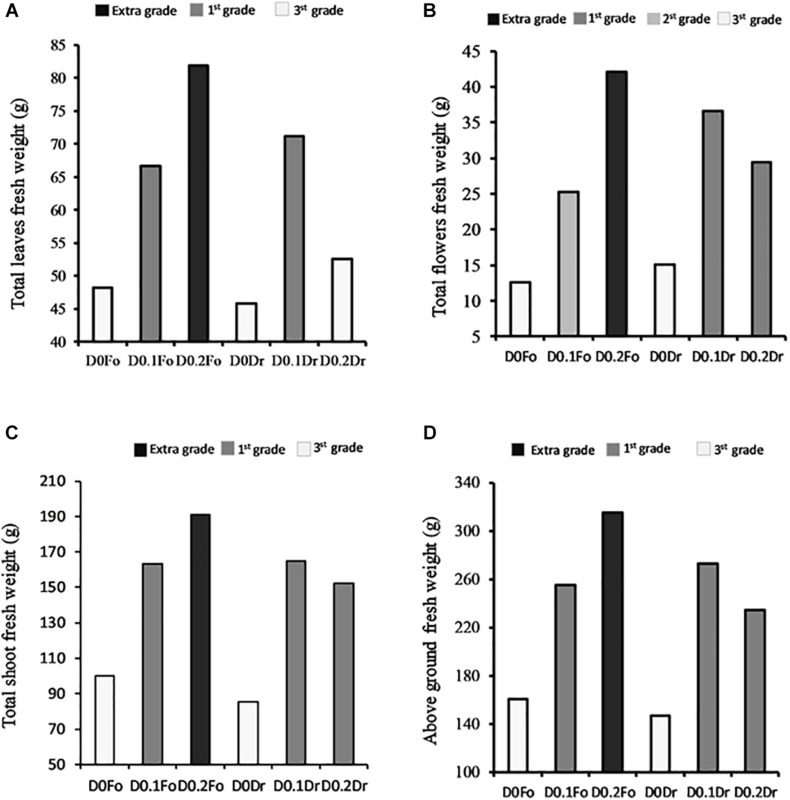
Interaction effects of biostimulant dose (D0, D0.1, and D0.2) × application method (Fo = foliar spray and Rd = root drenching) on petunia plant grade [total fresh weight of leaves **(A)**, total fresh weight of flowers **(B)**, total fresh weight of shoots **(C)**, and aboveground fresh weight **(D)**].

Figure 2 shows that plants treated with increasing concentrations of foliar spray from 0 to 0.1 and 0.2 g L^–1^ increased in quality grade: 3rd grade (control) < 1st grade (D0.1 g L^–1^) < extra grade (D0.2) in terms of shoots (+91%), flowers (+230%), and leaf fresh weight (+71%). With root drenching at D0.1 or D0.2, plants achieved 1st grade.

### Root Morphology

Application of the animal-derived PH biostimulant Hydrostim to the plants at doses of 0.1 and 0.2 g L^–1^, whether by foliar spray or root drenching, positively influenced root morphology with respect to untreated plants (Tables 3, 4). The D × M interaction effect of biostimulant application (D × M) was significant for root length, projected and total root surface area (Table 3), and number of root tips, forks, and crossings (Table 4).

**TABLE 3:** Dose × method interaction of animal-derived PH biostimulant on root length (m × 10^3^/plant), projected area (cm^2^/plant), total surface area (cm^2^/plant) in petunia plants for doses D0, D0.1, and D0.2, and foliar spray and root drench application methods.

Root parameters	Dose (g L^–^^1^) (D)					
	0		0.1		0.2	
	
	Application method					
	Foliar spray	Root drenching	Foliar spray	Root drenching	Foliar spray	Root drenching
Length	1.25d	1.33d	2.14c	2.66b	3.15a	2.74b
Projected area	66.6c	70.6c	91.2bc	111.1b	146.5a	138.4a
Total surface area	202.2d	220.1d	272.9cd	347.9bc	435.0a	414.7ab

*Different letters in a given row indicate significant differences according to Duncan multiple-range test (P ≤ 0.05) (n = 3).*

**TABLE 4:** Dose × method interaction of animal-derived PH biostimulant on root tips (n 10^3^/plant), forks (n 10^3^/plant), and crossings (n 10^3^/plant) in petunia plants for doses D0, D0.1, and D0.2 and foliar spray and root drench application methods.

Root parameters	Dose (g L^–^^1^) (D)					
	0		0.1		0.2	
	
	Application method					
	Foliar spray	Root drenching	Foliar spray	Root drenching	Foliar spray	Root drenching
Tips	8.4d	9.0d	18.4b	15.2c	24.3a	18.0b
Forks	7.1d	7.5d	15.5b	12.8c	20.4a	15.2b
Crossings	153.7c	167.3c	207.4bc	264.4ab	330.6a	314.5a

*Different letters in a given row indicate significant differences according to Duncan multiple-range test (P ≤ 0.05) (n = 3).*

Regarding root length and projected and total root surface area, plants treated by foliar spray at a dose of 0.2 g L^–1^ achieved higher values than plants treated differently: 3.15 m × 10^3^ plant^–1^, 146.5 cm^2^ plant^–1^, and 435.0 cm^2^ plant^–1^, respectively (Table 3). The same significant trend was recorded in plants treated with 0.2 g L^–1^ as foliar spray (Table 4) for number of root tips (24.3 × 10^3^ plant^–1^), forks (20.4 × 10^3^ plant^–1^), and crossings (330.6 × 10^3^ plant^–1^).

### Leaf Nutritional Status

The D × M interaction of biostimulant application was found to be significant for leaf content of macronutrients N, P, and K (Table 5). Doses D0.1 and D0.2 as a foliar spray both increased total N (+54 and +65%, respectively), whereas when the biostimulant was applied as root drench, N content increased by 43%. P and K content achieved higher values than untreated petunias, when plants were treated with D0.2 as foliar spray, increasing by +0.33 and +38%, respectively. The only significantly different leaf content of K was recorded with root drenching at D0.1: +3.08% with respect to control.

**TABLE 5:** Dose × method interaction of animal-derived PH biostimulant on N, P, and K leaf total content in petunia plants for doses D0, D0.1, and D0.2 and foliar spray and root drench application methods.

Leaf mineral content (%)	Dose (g L^–^^1^) (D)					
	0		0.1		0.2	
	
	Application method					
	Foliar spray	Root drenching	Foliar spray	Root drenching	Foliar spray	Root drenching
N	2.02c	2.10c	3.11ab	2.91b	3.34a	2.89b
P	0.12c	0.15c	0.23b	0.22b	0.32a	0.21b
K	2.36c	2.38c	2.97a	2.52b	3.27a	2.45b

*Different letters in a given row indicate significant differences according to Duncan multiple-range test (P ≤ 0.05) (n = 3).*

### SPAD, Leaf Gas Exchange, and Chlorophyll Fluorescent Measurements

Animal-based PH biostimulant had a positive influence on parameters related to SPAD, leaf gas exchange, and chlorophyll fluorescence (Table 6). The D × M interaction effect of biostimulant application (D × M) was significant for SPAD: using foliar spray at doses D0.1 and D0.2, SPAD showed a linear increase (+21.6 and +41.0%) with respect to untreated plants. Conversely, using root drenching, D0.1 produced an increase (+13%) with respect to control.

**TABLE 6:** Dose × method interaction of animal-derived PH biostimulant on chlorophyll index (SPAD), net photosynthesis (μmol CO_2_ m^–2^ s^–1^), stomatal conductance (mmol H_2_O m^–2^ s^–1^), and chlorophylls fluorescence (Fv/Fm) in petunia plants for doses D0, D0.1, and D0.2 and foliar spray and root drench application methods.

Physiological parameters	Dose (g L^–^^1^) (D)					
	0		0.1		0.2	
	
	Application method					
	Foliar spray	Root drenching	Foliar spray	Root drenching	Foliar spray	Root drenching
Chlorophyll Index	37.5c	37.7c	45.6b	42.6bc	52.9a	39.8c
Net photosynthesis	14.9c	17.2c	22.9a	21.5a	22.4a	19.6b
Stomatal conductance	0.25c	0.26c	0.39ab	0.37ab	0.42a	0.33b
Chlorophyll fluorescence	0.83c	0.82c	0.95a	0.91b	0.90b	0.89b

*Different letters in a given row indicate significant differences according to Duncan multiple-range test (P ≤ 0.05) (n = 3).*

Concerning leaf gas exchange parameters, application of biostimulant as foliar spray at both doses led to a significant improvement in net photosynthesis (D0.1: 22.9 μmol CO_2_ m^–2^ s^–1^; D0.2: 22.4 μmol CO_2_ m^–2^ s^–1^) and stomatal conductance (D0.1: 0.42 mmol H_2_O m^–2^ s^–1^ and D0.2: 0.39 mmol H_2_O m^–2^ s^–1^) with respect to control plants.

A significant D × M interaction was found for chlorophyll fluorescence: application of biostimulant as foliar spray at D0.1 was associated with the highest value (Table 6).

## Discussion

In this article, potted petunia plants were treated at two doses (D0.1 = normal and D0.2 = high) or not treated (D0) with an animal-based PH biostimulant, Hydrostim, applied by foliar spray or root drenching. The first dose of biostimulant D0.1 was that recommended by the manufacturer; the second D0.2 was double that amount.

Ertani et al. (2013) found that application of low doses (0.01 and 0.1 mL L^–1^) of an animal-derived PH, rich in amino acids, promoted maize seedling growth. In their experiments, other authors have applied the recommended dose: Casadesús et al. (2020) applied an animal PH biostimulant (Pepton) at a recommended dose of 4 kg ha^–1^ to tomato plants; Cristiano et al. (2018) treated snapdragon plants at a recommended dose of 0.1 g L^–1^. The effectiveness of the application method (foliar spray or soil drench) appears to be species-dependent: Sestili et al. (2018) showed that drench applications of PH were more effective in improving plant growth and total N uptake than foliar sprays in tomato.

In the present study, an animal-derived PH biostimulant (Hydrostim), rich in amino acids (52%), was applied to petunias and was found to improve visual quality traits (Figure 1), plant biomass (Figure 2), leaf nutrient content (Table 3), root morphology (Tables 4, 5), and leaf gas exchange (Table 6). Our findings show that the most promising treatment for petunia plants was foliar spray at 0.2 g L^–1^. On the contrary, Cerdán et al. (2008, 2013) observed growth inhibition in tomato plants treated with an animal-derived PH, and Lisiecka et al. (2011) found no improvement in strawberry plants. These different results could be due to the different commercial PHs, plant species, concentrations, and growth conditions.

Our visual quality trait results (Figure 1) agree with the findings of other researchers; for example, Zulfiqar et al. (2019) demonstrated that the biostimulant *Moringa oleifera* leaf extract (MOLE) improved preharvest quality of sword lily: corms soaked in MOLE + salicylic acid + gibberellic acid showed enhanced growth and development: longer floral stems, more leaves and larger leaf area. Parrado et al. (2008) demonstrated that application of an animal-derived PH (Siapton) to tomato plants elicited a significant increase in various plant growth parameters and number of flowers per plant. Ertani et al. (2013) obtained 4 and 8% increases in leaf dry weight of maize at meat-hydrolysate doses of 0.01 and at 0.1 mL L^–1^, respectively.

Regarding ornamental flower crops, the present study is in line with reports by De Lucia and Vecchietti (2012), who investigated the effects of three different agricultural biostimulants on lily hybrids grown in a soilless system: animal-derived PH biostimulant increased leaf area and flower bud number with respect to untreated controls. It is thought that signaling molecules in the biostimulant, such as free amino acids, promote endogenous phytohormonal biosynthesis, thus stimulating growth (Rouphael et al., 2017b).

Bulgari et al. (2015) found that bedding plant quality depended on visual appearance, as well as flower number and plant biomass. Cirillo et al. (2018) found that the effects on growth, ornamental quality, leaf gas exchanges, and mineral composition of spraying three different species of bedding plant (*Begonia tuberhybrida*, *Pelargonium peltatum*, and *Viola cornuta*) with increasing concentrations of a commercial legume-derived PH (Trainer) (0, 1, 3, and 5 mL L^–1^) were species-dependent. In particular, the normal concentration (1 mL L^–1^) enhanced several growth and quality parameters (plant height, canopy volume, leaf area, and number of flowers per plant) of *P. peltatum*, whereas positive effects of biostimulant application to *B. tuberhybrida* and *V. cornuta* were only observed at higher concentrations.

The results of our experiment demonstrate that Hydrostim, containing organic N and amino acids, has multifaceted action that may ensure achievement of extra-grade quality in Petunia (Figures 1, 2). Lucini et al. (2018) showed that substrate drench with a biostimulant containing lateral root–promoting peptides and lignosulfonates increased biomass production in melon. Cristiano et al. (2018) showed that aboveground plant biomass was not differentially affected by the method of application of biostimulant in two F1 *Antirrhinum majus* L. hybrids (“yellow floral showers” and “red sonnet”). By contrast, our results (Figures 1, 2), suggest that PH biostimulant applied to petunia as foliar spray increased fresh and dry biomass production more than drenching (Figure 2), mainly through slightly higher leaf area (larger surface for light assimilation, Figure 1) and SPAD and significantly higher net photosynthesis rate (Table 6). According to Rouphael et al. (2017a), increasing crop effectiveness is due to greater absorption of nutrients.

Our findings show that N content increased with application of PH biostimulant as foliar spray (Table 5). N content is also important regarding visual quality assessment of the leaves: discoloration such as yellowing appears in the older leaves and a size reduction of younger leaves due to N deficiencies too (Gibson et al., 2007; Barker and Pilbeam, 2007; Datnoff and Elmer, 2016). Flowering is in general delayed and reduced in number and size. Our hypothesis, based on the recent literature, is that higher N content could be related to increased gene expression. Wilson et al. (2018) reported that gelatin hydrolysate treatment increased the expression of genes coding for amino acid permeases (*AAP3* and *AAP6*) and transporters of amino acids and N. They concluded that gelatin hydrolysate provided a sustained source of N and acted as a biostimulant.

P and K are also important elements for plant growth, as well as visual and overall quality. P plays a significant role in energy storage, energy transfer, photosynthesis, cell division, and cell enlargement. Niedziela et al. (2008) showed that shorter stem length was due to P deficiency in *Lilium longiflorum*. Adequate P is needed for the promotion of early root formation and growth. K deficiency has been associated with fewer flowers (Dufault et al., 1990). In photosynthesis, K regulates the opening and closing of stomata and therefore CO_2_ uptake (Wang et al., 2013). It plays a major role in water regulation in plants (osmoregulation) and is essential at almost every step of protein synthesis. Colla et al. (2017a) showed that four foliar applications of a legume-derived PH at a concentration of 3 mL L^–1^ during the growing cycle increased K content of greenhouse tomatoes. Recent studies have shown yield and nutrient uptake enhancement with PH biostimulant (Calvo et al., 2014).

In our experiment, the better agronomic responses of PH-treated petunia may be associated with enhanced root morphology (Tables 4, 5) that could facilitate N uptake and leaf N content. In maize treated hydroponically with 0.01 and 0.1 mL L^–1^ of a meat-hydrolysate biostimulant, Ertani et al. (2013) found that root dry weight increased by +30 and +24%, respectively, compared to untreated controls. Casadesús et al. (2020) assessed the effect of Pepton (an animal-based PH biostimulant) on tomato plants cultivated under suboptimal conditions. They found that Pepton had a positive effect on primary and lateral root growth through a direct influence of amino acid availability and through salicylic acid accumulation in response to stressful conditions.

Our results agree with those of other studies that have shown that applications of plant- and animal-based PH biostimulants are able to optimize plant photosynthesis (Kang and van Lersel, 2004; Rouphael and Colla, 2020). The enhanced photosynthetic capacity observed in our petunias treated with Hydrostim as foliar spray increased biomass accumulation (Figure 2 and Table 3). Leaf chlorophyll content (SPAD index) was consistent with the results observed for photosynthetic activity. Loh et al. (2002) showed that SPAD is useful for assessing the quality of ornamentals, as it is correlated with good general condition and leaf greenness.

## Conclusion

In this article, we compared three doses (0, 0.1, and 0.2 g L^–1^) and two application methods (foliar spray and root drenching) to assess the effect of a commercial biostimulant (Hydrostim: animal-based PHs) on visual quality, biomass, macronutrient content, root morphology, and leaf gas exchange in potted Petunia cultivation. We found that application as foliar spray at a dose of 0.2 g L^–1^ helped to achieve extra-grade plants; the high dose (D0.2) also had the strongest effect on dry biomass; leaf N, P, and K content; and root morphology.

In the last 10 years, much attention has been focused on the use of biowaste-sourced products, such as animal-based PHs, in ecofriendly sustainable agriculture, due also to the contribution of these products to the problem of waste disposal. Our results suggest that application of animal-based PHs can be exploited under sustainable greenhouse conditions in the commercial production of petunia.

## Data Availability Statement

The raw data supporting the conclusions of this article will be made available by the authors, without undue reservation.

## Author Contributions

GC and BD conceived and designed the research, performed the experiments, prepared the materials, analyzed the data, and wrote the manuscript. Both authors revised the manuscript, read, and approved the final manuscript.

## Conflict of Interest

The authors declare that the research was conducted in the absence of any commercial or financial relationships that could be construed as a potential conflict of interest.

## Footnotes

1 https://www.flowerscanadagrowers.com/home
