# Supplementary material for: Petunia Performance Under Application of Animal-Based Protein Hydrolysates: Effects on Visual Quality, Biomass, Nutrient Content, Root Morphology, and Gas Exchange
Source: Front Plant Sci. 2021 Jun 14;12:640608. doi: 10.3389/fpls.2021.640608 (PMC8236847; doi:10.3389/fpls.2021.640608)
Supplement: Supplementary file 1 [file Image_1.pdf]

Petunia performance under application of animal-based protein hydrolysates: evaluation of visual quality, biomass, nutrient content, root morphology, and gas exchange

Giuseppe Cristiano and Barbara De Lucia Department of Agricultural and Environmental Sciences, University of Bari Aldo Moro, 70126 Bari, Italy

Sustainable plant production practices were studied to reduce the use of synthetic fertilizers and other agrochemicals. One way to reduce fertilizer use without damaging plant nutrition is to enhance crop uptake of nutrients with biostimulants.

Petunia requires high nutrient inputs to meet growth demands and these need to be supplied by the growing medium or fertilizers. Information is needed to optimize floricultural practices for petunia production with a view to improving quality and sustainability.

The aim of this research was to evaluate the effect of a commercial animal-based protein hydrolysate (PH) biostimulant on the visual quality, biomass, macronutrient content, root morphology and leaf gas exchange of a petunia (*Petunia* x *hybrida* Hort. 'Red').

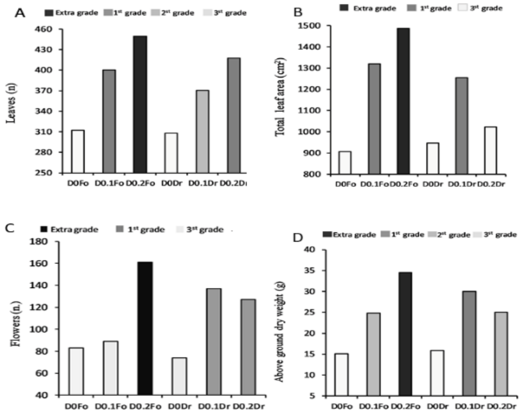

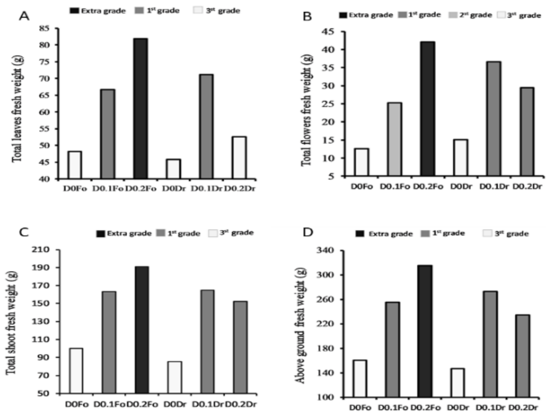

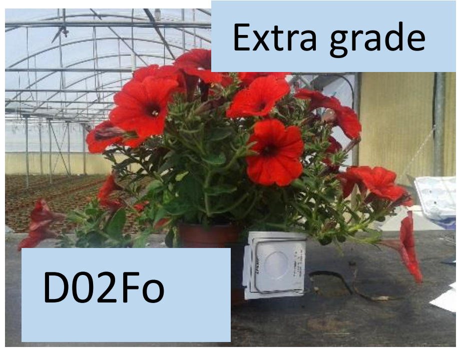

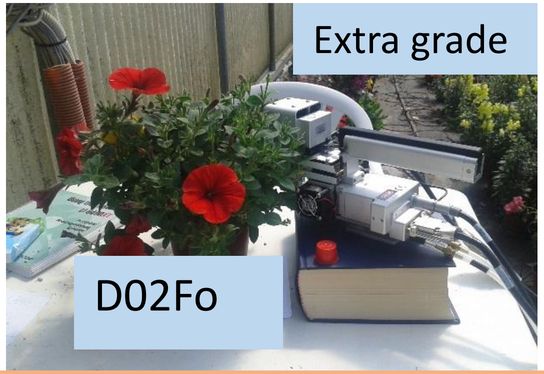

Quality has various extrinsic, or visual, and intrinsic, such as environmental and social, components. Consumers choose flowering plants with high aesthetic quality: compact, branched, with many flowers and leaves, a good balance between plant and pot size, and dark green leaves without blemishes or signs of stress.

We found that application as foliar spray at a dose of 0.2 gL^{-1} helped to achieve Extra grade plants; the higher dose (D0.2) also had the strongest effect on dry biomass, leaf NPK content and root morphology.
